# Unusual Magnetic Order in Eu_11–*x*_Hg_54+*x*_

**DOI:** 10.1021/acsorginorgau.5c00099

**Published:** 2025-12-22

**Authors:** Rachel Nixon, Nazar Zaremba, Samuel A. Adegboyega, Andreas Leithe-Jasper, Mitja Krnel, Yurii Prots, Lev Akselrud, Marcus Schmidt, Ulrich Burkhardt, Jörg Sichelschmidt, Lucia Amidani, Fabio La Mattina, Michael Shatruk, Alexander Shengelaya, Manuel Brando, Eteri Svanidze

**Affiliations:** † Max Planck Institute for Chemical Physics of Solids, 01187 Dresden, Germany; ‡ School of Chemistry, University of St Andrews, KY16 9ST St Andrews, U.K.; § Department of Chemistry and Biochemistry, 7823 Florida State University, Tallahassee, Florida 32306, United States; ∥ Ivan Franko Lviv National University, Lviv 79007, Ukraine; ⊥ 55553 The Rossendorf Beamline at ESRF—The European Synchrotron, 38000 Grenoble, France; # Institute of Resource Ecology, Helmholtz-Zentrum Dresden-Rossendorf (HZDR), 01328 Dresden, Germany; ¶ Laboratory for Transport at Nanoscale Interfaces, Empa Swiss Federal Laboratories for Science and Technology, 8600 Dübendorf, Switzerland; ∇ Department of Physics, 112482 Ivane Javakhishvili Tbilisi State University, Tbilisi 0173, Georgia; ○ Andronikashvili Institute of Physics, 112482 Ivane Javakhishvili Tbilisi State University, Tbilisi 0177, Georgia

**Keywords:** mercurides, complex magnetic structure, complex intermetallic compounds, spin texture, ferrimagnetism

## Abstract

In solid-state compounds, the valence of europium can sometimes be mixed, which is especially favored in structures with several positions for the europium atoms. In this work, we study the Eu-based intermetallic noncentrosymmetric system Eu_11–*x*_Hg_54+*x*_, which has 65 atoms per unit cell and 4 distinct crystallographic positions for europium and 14 positions for mercury. Our detailed analysis of the magnetism of large single crystals suggests that europium in Eu_11–*x*_Hg_54+*x*_ might be present in two valence states, resulting in a fragile magnetic ground state. Due to the cage-like structure with a large distance between the Eu atoms, those atoms are weakly ferromagnetically coupled and Eu_11–*x*_Hg_54+*x*_ orders at low temperatures, below *T*_1_ = 5.5 K, with a subsequent spin reorientation at *T*_2_ = 4.3 K. There is no sign of magnetic frustration. Interestingly, the magnetic ordering of the europium substructure results in a magnetization pole reversal with a delicate ferrimagnetic ground state. Additional magnetic phases can be induced by the application of a modest external magnetic field.

## Graphical abstract

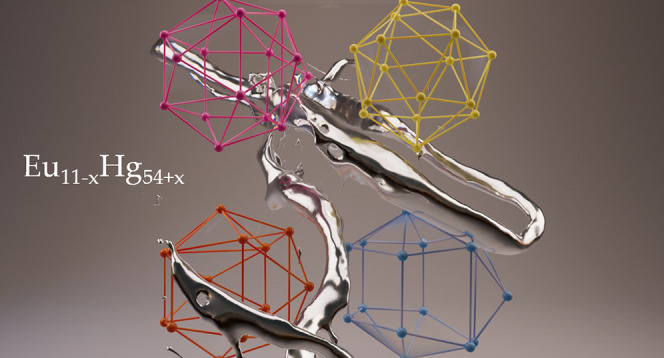

## Introduction

Intermediate valence and mixed valence compounds offer a great playground for correlated electron physics and unusual bonding phenomena, especially among rare-earth-based materials. Cerium (and the majority of other rare-earths) may have an oxidation state of +3 or +4, while samarium, europium, thulium, and ytterbium may have an oxidation state of +2 or +3. In fact, even among rare-earths, europium is the odd one out—it mostly exhibits the +2 rather than the +3 oxidation state,^1,2^ with the divalent oxidation states common in intermetallics and the trivalent prevalent in ionic materials. Furthermore, europium is significantly less abundant and has a lower melting temperature and density compared to its neighbors in the Periodic Table. The similar size and stability of Eu^2+^ cations explain the tendency of europium to substitute for Ca^2+^ in minerals,^3,4^ serving as a pertinent geochemical marker. The flexible oxidation state of europium is also the reason behind many peculiar low-temperature properties of quantum materials containing europium; in this sense, europium is similar to cerium, samarium, thulium, and ytterbium. While the sizes of the Eu^2+^ and Eu^3+^ ions differ (1.250 Å vs 1.066 Å for coordination of 8),^5^ the fast electronic fluctuations frequently make the coexistence of both species possible. Additionally, some structures can promote sites with different volumes of the coordination environment. Moreover, the mixed valence can be either static or dynamic, with the latter resulting in abrupt changes in structure and electronic properties. This has prompted many mixed-valent compounds containing europium to be investigated over the past decades.^6–24^

While mercury is frequently thought of in relation to superconductivity,^25–30^ its large spin–orbit coupling has been shown to promote the emergence of peculiar topological states.^31–36^ Associated experimental difficulties such as toxicity and air-sensitivity^37,38^ can be solved by utilizing a confined laboratory and adapted measurement methods.^39–45^ Recent synergetic efforts between chemical^37,46–59^ and physical^40–45^ investigations revealed that many new materials can be discovered—spanning a whole range of chemical complexity and ground states, ranging from magnetic to superconducting.

In this work, we present the magnetic properties of the noncentrosymmetric Eu_11–*x*_Hg_54+*x*_ compound. Its complex crystal structure lies at the origin of the intricate magnetic phase diagram that it displays. The average oxidation state of Eu ranges from +2.02 to +2.18, placing Eu_11–*x*_Hg_54+*x*_ in a family of inhomogeneous mixed-valence systems.^2^ Magnetic order occurs below 5.5 K. The coordination environment of Eu in Eu_11–*x*_Hg_54+*x*_ consists of 14–16 atoms, typical of rare-earth systems. This links Eu_11–*x*_Hg_54+*x*_ to magnetically ordered cluster-like compounds such as EuCd_11_ and UCd_11_ (CN = 20, AFM below *T*_N_ = 2.7 K^60,61^ and 5 K,^62–65^ respectively), U_23_Hg_88_ (CN = 14–16, AFM below *T*_N_ = 2.2 K^40^), and U_2_Zn_17_ (CN = 19, AFM below *T*_N_ = 9.7 K^65^).

## Experimental Methods

Many issues arise from experimental work with mercury including high vapor pressure, high chemical reactivity, toxicity, and extreme air sensitivity. To mitigate these effects, a specialized laboratory environment is needed.^39^ As we have previously shown,^40–43^ it is possible to obtain intrinsic crystallographic and physical property data on mercurides and amalgams. Five samples were synthesized by combining Hg (droplet, Alfa Aesar, 99.999%) with Eu (pieces, Alfa Aesar, 99.9%) with a Eu:Hg ratio of 5:95. Samples were sealed in tantalum tubes to prevent the evaporation of constituent elements. To protect samples from air and moisture, all syntheses were performed in an argon-filled glovebox system. The synthetic profile consisted of heating to 500 °C and then slowly cooling to room temperature over the course of 7 days. The samples were then placed in specialized crucibles^66^ and centrifuged at room temperature to remove the excess mercury. This method can eliminate most mercury; any remainder can be removed by allowing the crystals to sit on gold foil for several days.

Details on sample preparation and data analysis for powder X-ray diffraction, electron spin resonance (ESR), magnetic properties, X-ray absorption measurements, and thermal analysis can be found in the Supporting Information.

## Analysis of the Crystal Structure and Magnetic Properties

Among europium-based materials that have been reported to crystallize in the noncentrosymmetric *P*6̅ space group, Eu_2_(Ni/Co)_12_P_7_ ^67,68^ (Zr_2_Fe_12_P_7_ structure type) and EuNaF_4_ ^69^ (NaNdF_4_ structure type) display trivalent europium. Divalent europium has been reported for the Eu_2_Mg_3_Cu_9_(As/P)_7_ ^70^ and Eu_2_Yb_1.11_Mg_10.89_Si_7_ ^71^ compounds (both Zr_2_Fe_12_P_7_ structure type) as well as Eu_5_In_9_Pt_7_,^72^ EuBa_6_Cl_2_F_12_,^73^ and Eu_7_Cl_2_F_12_ ^74^ (all Ba_7_Cl_2_F_12_ structure type). The formation of the mercurides with the 11:54 stoichiometry has so far only been reported for Na,^57,75^ Ca,^59^ Sr,^45,59^ Eu,^48^ and Yb.^76^ Given the similarity of Eu^3+^ (1.06) and Eu^2+^ (1.25 Å) to Ca^2+^ (1.12 Å) and Sr^2+^ (1.26 Å) (see Figure 1a), it is likely that the homogeneity ranges for the three isostructural systems would be comparable.[Fn fn1] Nonetheless, to investigate the variation of stoichiometry and as a result of the magnetic properties in the Eu_11–*x*_Hg_54+*x*_ system, five samples have been prepared (see Supporting Information).

**Figure 1:**
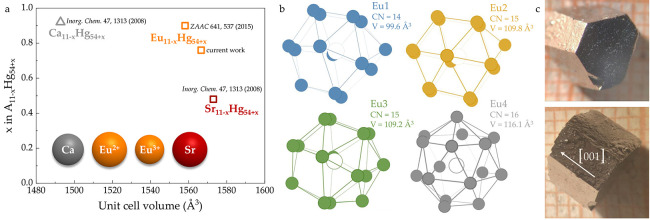
(a) Small variations in the volumes of the A_11–*x*_Hg_54+*x*_ (A = Ca, Sr, or Eu) family are driven by mixed occupancy of the A4 position.^45,48,59^ The relative sizes of Ca, Sr, and Eu are shown in the inset. For superconducting A = Ca and Sr (gray and maroon, respectively), these defects do not change the value of *T*_c_ much (∼10% for *x* ≤ 0.5 ^44,45^ ). The data are taken from single-crystal X-ray diffraction refinements.^48,59^ (b) The complex magnetism of Eu_11–*x*_Hg_54+*x*_ is driven by the four distinct crystallographic sites of Eu. The coordination of Eu varies between 14 (Eu1), 15 (Eu2 and Eu3), and 16 (Eu4). (c) Single crystals of Eu_11–*x*_Hg_54+*x*_, placed on mm-paper.

The crystal structure of Eu_11–*x*_Hg_54+*x*_ was re-examined using single crystal diffraction. Due to the enormous absorption of the investigated material (μ = 143 mm^–1^ for MoKα radiation), only a small, irregular shaped piece (∼30 μm) was cut from the grown single crystal. The corresponding crystallographic data and atomic coordinates are listed in Tables S1 and S2 in the Supporting Information. Eu_11–*x*_Hg_54+*x*_ crystallizes in the noncentrosymmetric space group *P*6̅ and adopts the Ca_11–*x*_Hg_54+*x*_ structure type,^59^ related to Gd_14_Ag_54_.^77^ In contrast to earlier studies,^48^ the mercury subcell is completely ordered and described by 14 positions, instead of 16. The homonuclear Hg–Hg contacts cover a wide range between 2.859(3) and 3.547(2) Å. Comparing these values with the interatomic distances of 2.993 Å in the α-modification of the elemental (solid) mercury,^78^ it can be assumed that the Hg–Hg interactions vary from very strong to rather weak. Europium atoms are distributed across four crystallographic sites: Three 3-fold and one 2-fold positions. Similar to previous work,^48^ the latter is mixed with Eu and Hg atoms in a ratio of 0.62(3):0.38. Taking this fact into account, the formula for the reported phase should be written as Eu_11–*x*_Hg_54+*x*_ (*x* = 0.76 for Sample 1). Unfortunately, we were not able to determine accurate *x* values for all of the samples of this study—for Samples 2–5, the Eu_10_Hg_55_ composition is used.[Fn fn2]

The saturated magnetic moment expected for a purely Eu^2+^ compound should amount to 7 μ_B_. As can be seen from Figure 2a, all Eu_11–*x*_Hg_54+*x*_ samples show saturation of the *M*(*H*) isotherm, albeit with a smaller value of the moment per Eu.[Fn fn3] As mentioned above, for this work, 5 samples of Eu_11–*x*_Hg_54+*x*_ were synthesized and studied. Based on the value of μ (μ_o_ *H*= 7 T, *T* = 2 K), it is possible to estimate the ratio of Eu^3+^ to Eu^2+^ for each of the Eu_11–*x*_Hg_54+*x*_ samples, as summarized in Figure 2a. The average Eu valence, listed for each of the samples, ranges from 2.02 (pink) to 2.18 (orange) and is calculated as the average valence for the observed magnetic saturation. It is important to note that while the low-field data shows some anisotropy (dark vs light yellow of the inset), the saturated magnetic moment is isotropic. All samples exhibit Curie–Weiss behavior. By fitting the inverse susceptibility above *T* = 100 K, a positive Weiss temperature θ_W_ = 6.4–11.7 K is extracted, indicating a ferromagnetic exchange interaction between the nearest-neighbor Eu atoms. In fact, the ordering temperature of Eu_11–*x*_Hg_54+*x*_ is about 5.5 K, but the ordered state is likely not purely ferromagnetic. This is not a surprise because the Eu atoms are in the +2 state, with *L* = 0 and *S* = 7/2, which means isotropic moments with no effect of the crystalline electrical field because of the missing orbital moment. This implies that a secondary weak antiferromagnetic interaction can still drive the system to be antiferromagnetic. This is reminiscent of the EuCd_2_P_2_ system,^20,79–82^ in which the relevant energy scale is ferromagnetic (positive θ_W_), and therefore, the Eu moments align ferromagnetically within the plane of the tetragonal structure, but an antiferromagnetic coupling between the planes drives the system to an overall antiferromagnetic ordering. The value of the effective fluctuating moment μ_eff_ = 7.24–7.85 μ_B_, extracted from the Curie–Weiss fits, is comparable to the calculated value for Eu^2+^ of 7.94 μ_B_; however, it is reduced due to the mixed valence state. As summarized in Figure 2b, magnetic isotherms show a number of features, which can be used to construct a preliminary phase diagram; see Figure 5a below. We have included all features, even though they might be not a signature of phase transitions. Of course, a more detailed understanding of the magnetic structure in Eu_11–*x*_Hg_54+*x*_ would be desirable; however, neutron scattering experiments, due to the large neutron cross sections of constituent elements, have not been carried out. It is, however, of interest to study this compound by ^151^Eu–Mössbauer spectroscopy and Eu L-edge X-ray magnetic circular dichroism spectroscopy, which should shed more light on this issue.^16,17,23,83^

**Figure 2:**
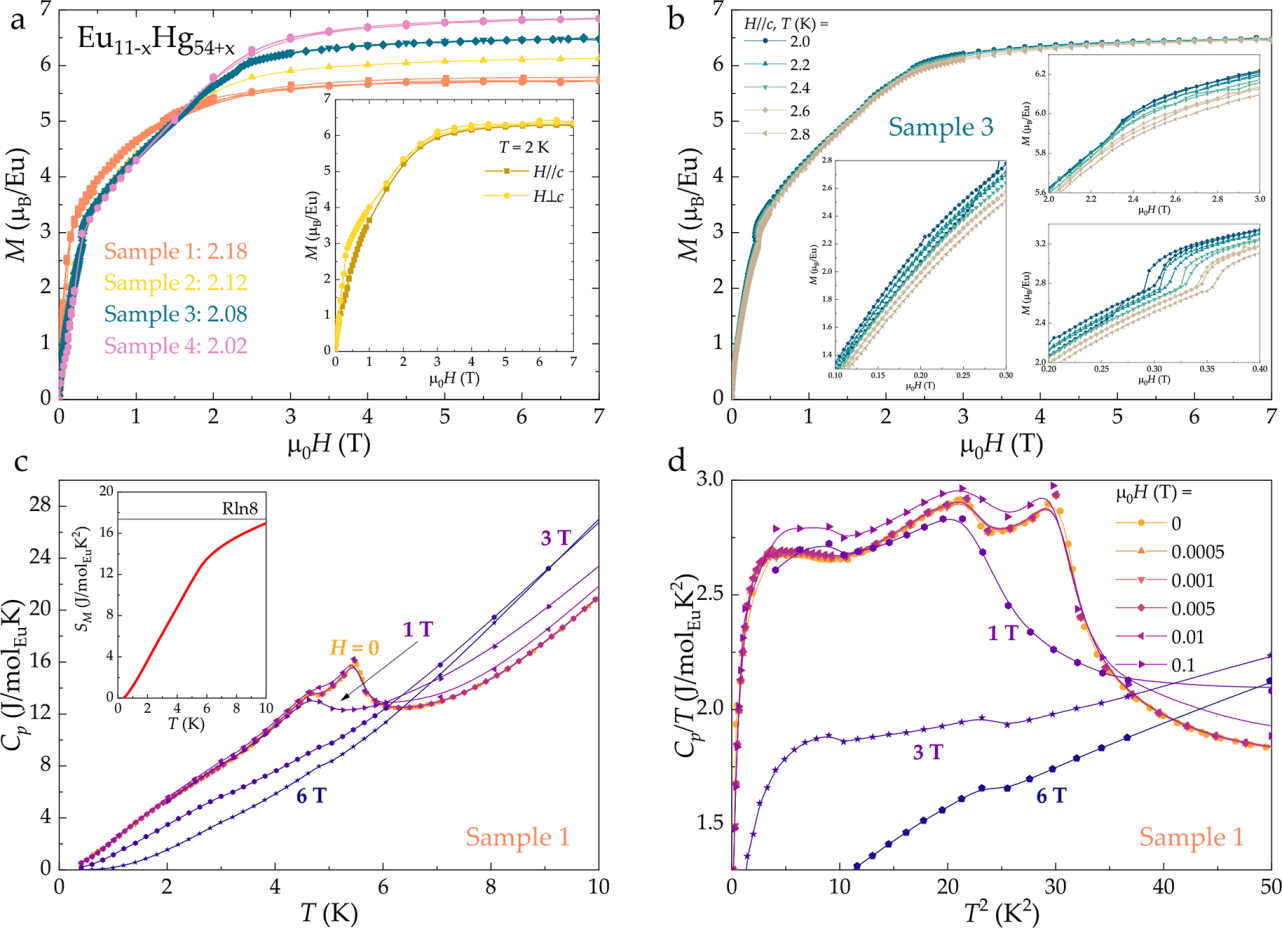
Magnetic properties of Eu_11–*x*_Hg_54+*x*_. (a) Isotherms, taken at *T* = 2 K, saturate with a moment less than that expected for a purely Eu^2+^ material (μ_sat,theory_ = 7 μ_B_). The value of saturated moment appears to be isotropic (see inset). Based on the value at μ_0_ *H* = 7 T, it is possible to estimate the relative ratio of Eu^2+^ to Eu^3+^, producing the average valence between 2.02 and 2.18. (b) Temperature-dependent isotherms show a number of features (insets), which are used to construct the *H*–*T* phase diagram. (c) Entrance into the magnetically ordered state below *T* = 5.5 K is marked by a sharp anomaly, which is gradually suppressed with magnetic field. The inset shows the entropy, reaching *R* ln 8 at *T* = 10 K. (d) The features, corresponding to transitions between different magnetic configurations are easier to track from the *C*_p_/*T* vs *T* ^2^ data.

The entrance into the magnetically ordered state below 5.5 K is also supported by the specific heat data of Eu_11–*x*_Hg_54+*x*_, shown in Figure 2c (Sample 1). As seen in the inset, full *R* ln 8 is only recovered at *T* = 10 K, meaning that magnetic fluctuations persist above the ordering temperature. This makes determination of the Sommerfeld coefficient γ not possible for Eu_11–*x*_Hg_54+*x*_. The structural similarity of Eu_11–*x*_Hg_54+*x*_ to U_23_Hg_88_,^40^ both of which are cluster-like structures, suggests that the possibility of effective mass enhancement in the former system should be investigated in more detail in the future.

The ordered state of Eu_11–*x*_Hg_54+*x*_ is unusual and fragile. This is demonstrated by a series of zero-field-cooled (zfc) and field-cooled (fc) measurements of the magnetization taken at very small magnetic fields. Selected data are shown in Figure 3. Because of the very small fields used and the presence of remnant field in the superconducting magnet (a few milli-Tesla), the absolute values of the susceptibility are not precise. In the fc data with *B* = 0.005 T, a very peculiar behavior is observed: First magnetization shows a small increase at *T*_1_ ≈ 4.98 K and then a strong decrease below *T*_2_ = 4.71 K to negative values. In the zfc data, the magnetic response is opposite and very symmetric. This is reminiscent of the magnetization pole reversal of ferrimagnetic systems like Ni­(HCOO)_2_·H_2_O,^84^ Mn_3–*x*_Ni_*x*_BO_5_ ^85^, or Pr_3_Fe_3_Sb_7_.^86^ This can be described by a model that considers two antiferromagnetically interacting subsystems, each being ferromagnetically ordered. At a slightly larger field of 0.01 T, we observe the same behavior, but the field-polarized component is larger. Small kinks and weak hysteresis are present at fields up to 0.35 T, as shown in Figure 3.

**Figure 3:**
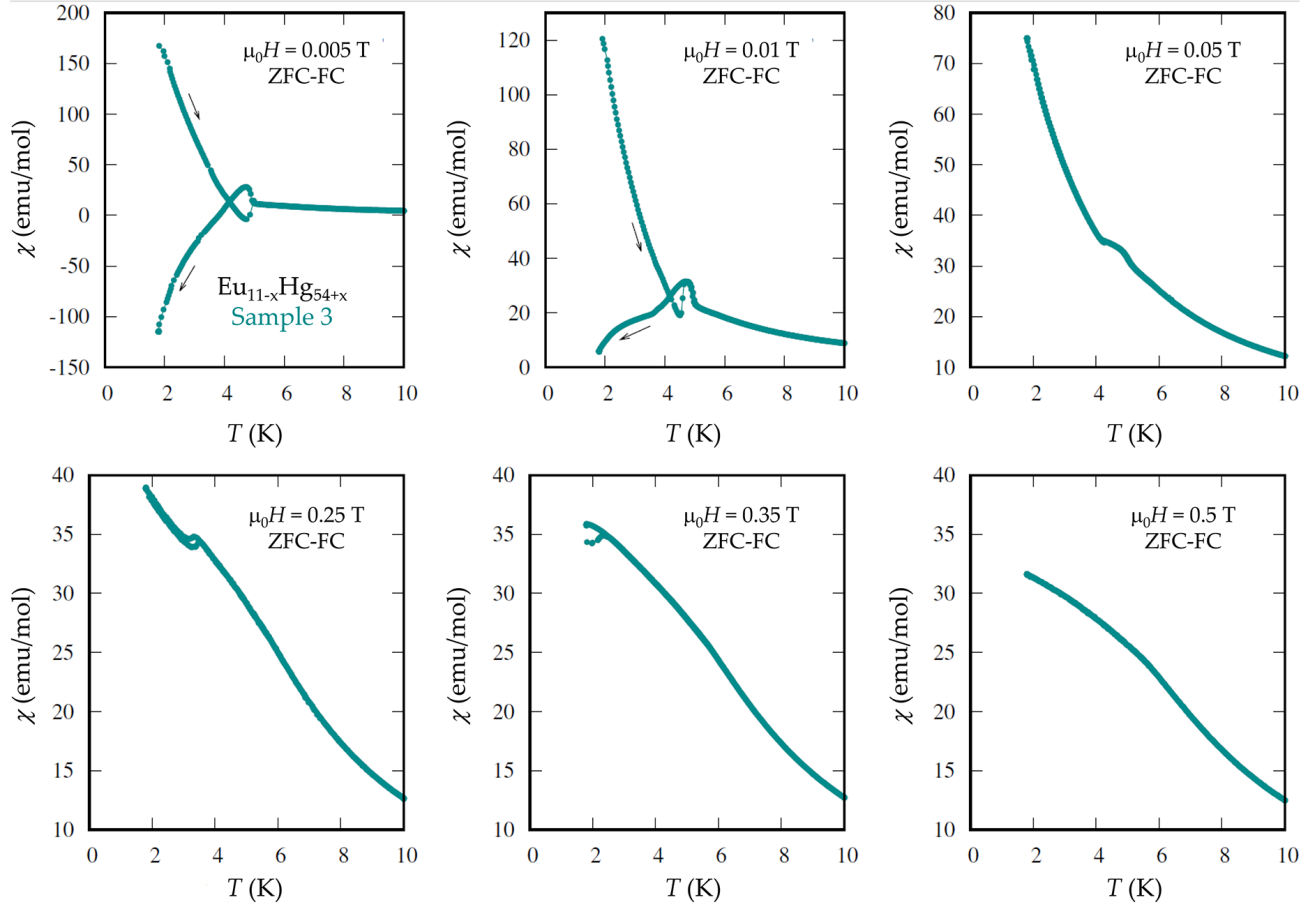
Zero-field-cooled (zfc) and field-cooled (fc) measurements of the magnetization of Eu_11–*x*_Hg_54+*x*_ (Sample 3) for six selected fields with *H*∥*c*. The magnetization pole reversal, observed at the lowest field of 0.005 T, has only been observed in a handful of systems.

To characterize the magnetic properties of Eu in Eu_11–*x*_Hg_54+*x*_ further, we employed the Electron Spin Resonance (ESR) as a local probe technique. In general, a Eu^2+^ ion in the magnetic 4f^7^ configuration (*L* = 0, *S* = *J* = 7/2) can be easily detected by ESR, whereas a Eu^3+^ ion with a paramagnetic (*J* = 0) 4f^6^ configuration is expected to be ESR-silent.^87–89^ Typical ESR spectra of Eu_11–*x*_Hg_54+*x*_ are shown in Figure 4a and c for selected temperatures. We have investigated the ESR of a single crystal as well as a powder sample, both originating from the same batch (Sample 3), in two separate temperature regions. It turned out that single-crystal ESR data are meaningful only at low temperatures, *T* < 10 K, where the microwave penetration depth is large enough, allowing the intensity of the sample signal to exceed that of the background signal. The anisotropy upon rotating the crystal in the external magnetic field was very weak, showing no resolvable changes in the line width and resonance field. Above 20 K, the ESR examination of powder ensures that, despite high electrical conductivity (small microwave penetration depth), most of the sample volume contributes to the ESR, resulting in a stronger ESR signal. Then, the ESR line was visible up to ≃50 K, above which the line broadening combined with the decrease of the spectra amplitude made it difficult to reliably fit the ESR spectra.

**Figure 4:**
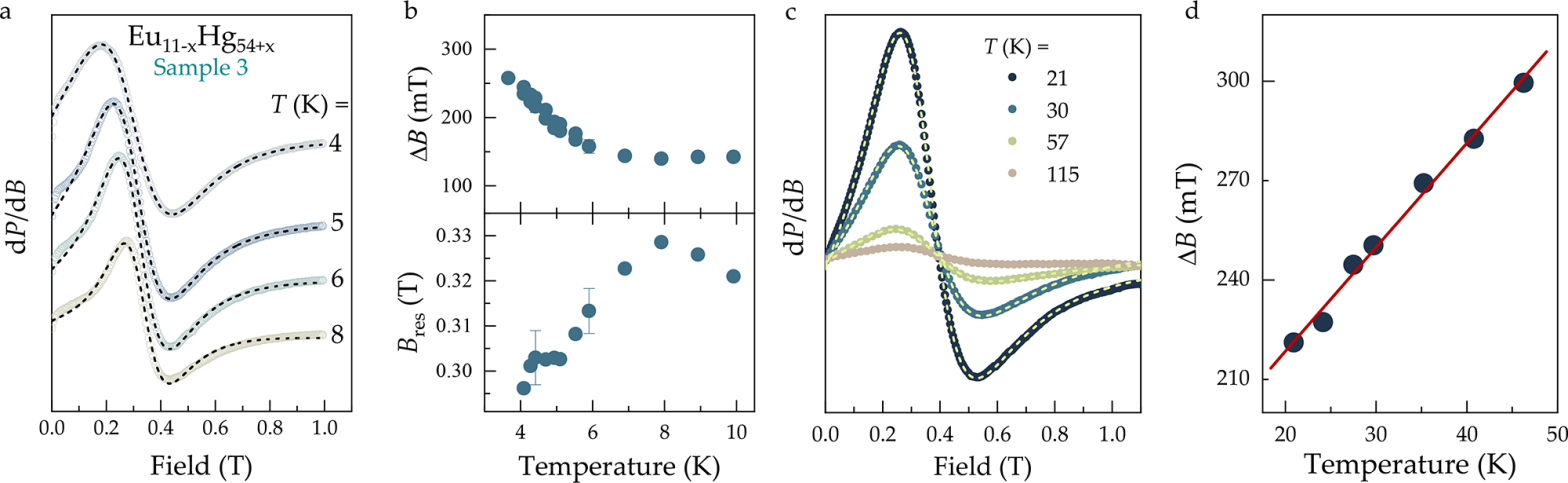
ESR spectra d*P*/d*B* (symbols) of Eu_11–*x*_Hg_54+*x*_ at different temperatures and Lorentzian line fittings (dashed lines) resulting in ESR line width Δ*B* and resonance field *B*_res_. ESR results shown in (a,b) refer to single crystalline and (c,d) to powdered Eu_11–*x*_Hg_54+*x*_ from Sample 3. Solid line shown in (d) represents the best fit to eq 1.

The results of the single-crystal ESR investigation are compiled in Figure 4a and b and those of the powder in Figure 4c and d. The best fit of the spectra to a single, asymmetric Lorentzian (”Dysonian”) line (see Supporting Information) is indicated by the dashed lines revealing an asymmetry that is increasing with decreasing temperature due to a reduced skin depth or increased sample conductivity toward low temperatures. This fitting yields the parameters, which are given in Figure 4b and d. As shown in Figure 4b, in the low-temperature region, the line width Δ*B* shows a continuous broadening below *T* ≃ 6 K, indicating the enhancement of Eu^2+^ spin correlations when approaching magnetically ordered phases below 5 K. At the same time, the resonance field *B*_res_ shows a pronounced temperature dependence. This points to the buildup of internal fields, as expected in the vicinity of magnetic order.

For *T* > 10 K, the resonance field *B*_res_ has a weak temperature dependence and slightly increases with increasing temperature. The obtained value of the *g* factor at 40 K, *g* = 2.01, is close to the value *g*_0_ = 1.9935, expected for an Eu^2+^ ion in a crystalline field environment of cubic symmetry.^90^ This suggests that the observed ESR spectra are due to the localized magnetic moments of Eu^2+^ ions in Eu_11–*x*_Hg_54+*x*_. The ESR intensity is proportional to the magnetic susceptibility of the ions, which produce the ESR signal. In Eu_11–*x*_Hg_54+*x*_, the dominant contribution to magnetic susceptibility is due to Eu^2+^ ions (see the discussion above). The ESR intensity shows a Curie–Weiss-like behavior, qualitatively similar to the bulk magnetic susceptibility data. This confirms that the observed ESR signal in Eu_11–*x*_Hg_54+*x*_ comes from the localized magnetic moments of Eu^2+^ ions. The ESR line width Δ*B* provides information on the local spin dynamics of the resonant magnetic moments. In this respect, important information is obtained from the temperature dependence of the line width plotted in Figure 4d, showing a linear thermal broadening. This indicates the dominant role of a Korringa relaxation of the localized Eu^2+^ moments via scattering off the conduction electrons

1 $$\Delta B\left(T\right)=\frac{\pi k_{B}}{g\mu_{B}}{\left(J_{fce}N\left(E_{F}\right)\right)}^{2}T=bT$$

where *J*_fce_ is the exchange constant between the Eu^2+^ 4f localized magnetic moments and the conduction electrons, *N*(*E*_F_) is the conduction electron density of states at Fermi Energy, and *b* is the Korringa slope.^91^ The obtained value in Eu_11–*x*_Hg_54+*x*_ is *b* = 3 mT/K, which is significantly larger than the typical value of *b* ≈ 1 mT/K of the *S* state 4f^7^ local moments in conventional metals.^91^ According to eq 1, the large Korringa slope in Eu_11–*x*_Hg_54+*x*_ indicates a strong coupling of the Eu^2+^ localized magnetic moments with conduction electrons or a large density of state at the Fermi level.

It is well-known that applied pressure tends to suppress the Eu^2+^ state in favor of the smaller-volume Eu^3+^ state.^92,93^ To explore such a possibility, we probed the magnetic properties of Eu_11–*x*_Hg_54+*x*_ (Sample 5) under applied pressure in a diamond anvil cell (DAC). A few small crystals of the material were loaded into the DAC, with the culet diameter of 0.5 mm. Small amounts of ruby and Nujol oil were added as pressure indicator and pressure-transmitting medium, respectively. The pressure was applied as force measured in kN, while the actual pressure was determined by measuring a shift in the ruby fluorescence peak: $P\left(GPa\right)=175.2\left[{\left(\lambda /\lambda_{0}\right)}^{10.7}-1\right]$ ,^94^ where λ and λ_0_ are wavelengths of maximum fluorescence observed under applied and ambient pressure, respectively. The magnetization data were corrected by subtracting the background measured on an empty DAC. The field-dependent magnetization measured at 10 K under ambient pressure and at 12.8 GPa did not reach saturation and did not show any anomalies, as expected for paramagnetic behavior above the ordering temperature. A comparison of the magnetization data normalized against the ambient-pressure curve (Figure 5c) revealed ∼5% decrease in the maximum magnetization at 7 T, suggesting that the applied pressure results in partial suppression of the Eu^2+^ state and increase in the average oxidation state of Eu. More detailed studies are planned in the future, combining magnetic measurements and X-ray Absorption Near Edge Structure (XANES) spectroscopy to understand the pressure-dependent electronic and magnetic behaviors of Eu_11–*x*_Hg_54+*x*_.

**Figure 5:**
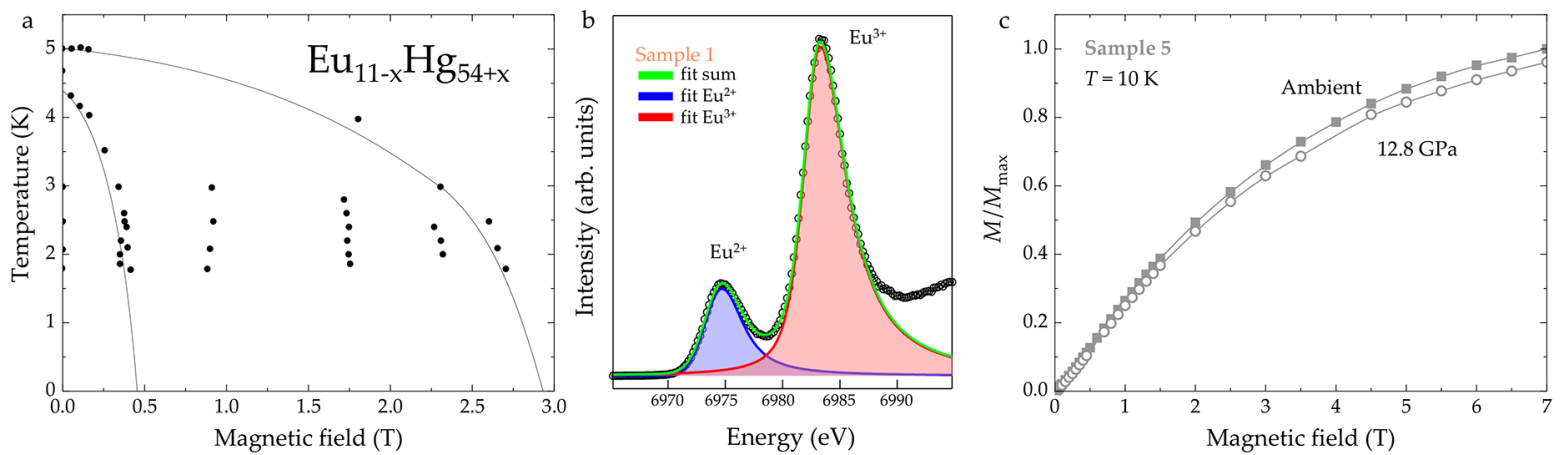
(a) The *H*–*T* phase diagram of Eu_11–*x*_Hg_54+*x*_ is likely driven by different sites of Eu. Within this phase diagram, the definitive assignment of magnetic configurations is not yet possible. (b) The XANES analysis of Sample 1 indicates that both Eu^2+^ and Eu^3+^ are present in Eu_11–*x*_Hg_54+*x*_. However, the relative ratios are not quantitative, given that Eu_11–*x*_Hg_54+*x*_ decomposes into Hg and Eu_2_O_3_ (in which Eu is in the 3+ state) during the measurement. (c) Evolution of the magnetic moment under ambient (black) and 12.8 GPa (red) pressure for Sample 5.

Ambient-pressure XANES at the Eu L_3_-edge was used to probe the oxidation state of Eu in Eu_11–*x*_Hg_54+*x*_ (Sample 1). The intense peak at the onset of the absorption, referred to as the white line, is found at 6975 eV for Eu^2+^ and at 6983 eV for Eu^3+^, making the two oxidation states easily distinguishable.^95^ The use of the High-Energy-Resolution Fluorescence-Detected (HERFD) mode to collect XANES results in sharper spectral features and increases considerably the sensitivity to the oxidation states of Eu.^96^ The HERFD XANES at the Eu L_3_-edge was collected on sample 1 at the ROBL^97,98^ beamline at the ESRF (Figure 5b). The sample was manipulated and mounted in the sample holder in an Ar glovebox. However, the Kapton window in front of the sample-holder used to allow X-rays to enter and exit was not sufficient to prevent air from penetrating. In the HERFD XANES spectrum, shown in Figure 5b, the peak corresponding to Eu^3+^ is dominating over the one of Eu^2+^. This quantitative discrepancy can be attributed to sample decomposition, described above.

## Conclusions

In this work, we present the analysis of the complex magnetic order of Eu_11–*x*_Hg_54+*x*_, which has four distinct crystallographic positions for Eu atoms. It appears that in this material, both Eu^2+^ and Eu^3+^ species are likely present, which is made possible by the cage-like structure of this compound. The magnetic order of Eu_11–*x*_Hg_54+*x*_ is fairly fragile, probably also a result of the structure, in which Eu moments are diluted. The exact magnetic structure assignment is hampered by the fact that both neutron diffraction studies and theoretical analysis are likely impossible for Eu_11–*x*_Hg_54+*x*_. Given that the isostructural Ca_11–*x*_Hg_54+*x*_ and Sr_11–*x*_Hg_54+*x*_ compounds exist and show superconductivity,^44^ partial substitution of Eu by either Ca or Sr is of interest and is currently being pursued. If an analysis of spin textures in Eu_11–*x*_Hg_54+*x*_ can be carried out in an air-free atmosphere, then this would also be of interest.

## Supplementary Material



## Data Availability

The data underlying this study are available in the published article and its Supporting Information.

## Footnotes

a This comparison does not include the Na and Yb analogues, since for those only lattice parameters refined from the single crystal diffraction experiments have been reported.^57,75,76^ Our preliminary work on these systems indicate slightly different values for the volume, which are still under investigation.^44^

b Since magnetic data are always scaled per Eu atom, this, in principle, does not affect the results of our analysis strongly. For example, for Sample 1, the value of the saturated magnetic moment μ (μ_0_ *H* = 7 T, *T* = 2 K) changes from 5.73 μ_B_ (*x* = 1) to 5.61 μ_B_ (*x* = 0.76).

c It is important to note that mixing of Eu and Hg on the Eu4 position can, in principle, produce compounds with more or less Eu, compared to the 11:54 ratio.^48^ However, to achieve a full 7 μ_B_ moment, a stoichiometry of Eu_8_Hg_57_ is needed (for Sample 1, for example), which would mean that the homogeneity range of this phase is far beyond that reported for other R/A_11–*x*_Hg_54+*x*_ phases—see Figure 1a. For the rest of the analysis, we therefore use the Eu_11–*x*_Hg_54+*x*_ formula, since the magnetic behavior of this phase is driven by the ratio of the Eu^3+^ to Eu^2+^ species rather than by the ratio of Eu to Hg.

## Notes

Synthesis details, electron spin resonance (ESR) analysis, analysis of magnetic properties, X-ray absorption measurements, thermal analysis, and single-crystal data (PDF)
