# Supplementary material for: Unusual Magnetic Order in Eu11–x Hg54+x
Source: ACS Org Inorg Au. 2025 Dec 22;6(1):130–8. doi: 10.1021/acsorginorgau.5c00099 (PMC12879176; doi:10.1021/acsorginorgau.5c00099)
Supplement: Supplementary file 1 [file gg5c00099_si_001.pdf]

Unusual magnetic order in $Eu_{11-x}Hg_{54+x}$

Rachel Nixon,[†,‡] Nazar Zaremba,[†] Samuel A. Adegboyega,[¶] Andreas Leithe-Jasper,[†] Mitja Krnel,[†] Yurii Prots,[†] Lev Akselrud,[§] Marcus Schmidt,[†] Ulrich Burkhardt,[†] Jörg Sichelschmidt,[†] Lucia Amidani,[‖,⊥] Fabio La Mattina,[#] Michael Shatruk,[¶] Alexander Shengelaya,[@,△] Manuel Brando,[†] and Eteri Svanidze[*,†]

†*Max Planck Institute for Chemical Physics of Solids, 01187 Dresden, Germany*

‡*School of Chemistry, University of St Andrews, St Andrews, KY16 9ST, United Kingdom*

¶*Department of Chemistry and Biochemistry, Florida State University, Tallahassee, FL 32306, United States*

§*Ivan Franko Lviv National University, Lviv, 79007, Ukraine*

‖*The Rossendorf Beamline at ESRF – The European Synchrotron, 38000 Grenoble, France*

⊥*Institute of Resource Ecology, Helmholtz-Zentrum Dresden-Rossendorf (HZDR), 01328 Dresden, Germany*

#*Laboratory for Transport at Nanoscale Interfaces, Empa Swiss Federal Laboratories for Science and Technology, 8600 Dübendorf, Switzerland*

@*Department of Physics, Ivane Javakhishvili Tbilisi State University, Tbilisi 0173, Georgia*

△*Andronikashvili Institute of Physics, Ivane Javakhishvili Tbilisi State University, Tbilisi 0177, Georgia*

E-mail: svanidze@cpfs.mpg.de

Supporting Information

X-ray diffraction

Powder X-ray diffraction was performed on a Huber G670 Image plate Guinier camera with a Ge-monochromator (CuKα_1 radiation, $\lambda = 1.54056$ Å) using LaB_6 as an internal standard. Powders were sealed between two Kapton films to prevent oxidation and decomposition. Phase identification was done using the Match 3! software.[1] The values of the unit cell volume for Samples 2-5, refined from powder X-ray data, were 1557.6 ± 0.7 Å^3.

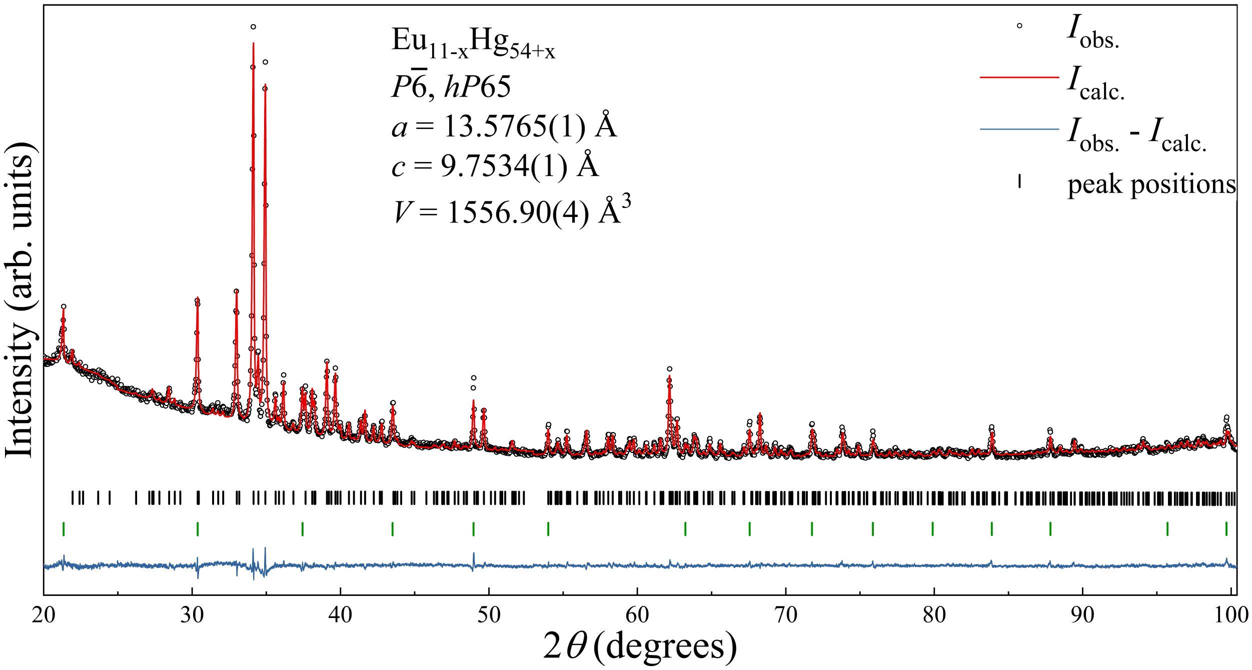

Figure S1: Powder XRD pattern for $Eu_{11-x}Hg_{54+x}$ (Sample 5) with LaB_6 as an internal standard. $Eu_{11-x}Hg_{54+x}$ phase is marked as black ticks, whereas LaB_6 is marked as green.

Electron spin resonance (ESR) analysis

In our ESR measurements we used a single crystal (arbitrarily oriented) and powder of $Eu_{11-x}Hg_{54+x}$ from the same batch (Sample 3). Both samples were mounted under Ar gas atmosphere in quartz sample tubes and fixed by paraffin. We used a continuous-wave ESR setup at X-band frequency ($\nu = 9.4$ GHz) equipped with a continuous He gas-flow cryostat

in the temperature range $3 < T < 300$ K. The resonance line was recorded as the field derivative of the absorbed microwave power P to enable an improvement of the signal to noise ratio by a lock-in phase sensitive detection. The resonance field (B_{res}, determined by the Eu g value and internal magnetic fields), linewidth (ΔB, determined by the Eu spin dynamics) and intensity (being a measure of the local susceptibility of the Eu spins) of the ESR signals were obtained from fitting the $\mathrm{d}P/\mathrm{d}B$ spectra with the first derivative of a Lorentzian shape:[2,3]

$$\frac{\mathrm{d}P}{\mathrm{d}B} \sim \frac{\mathrm{d}}{\mathrm{d}B}\left[\frac{\Delta B + \alpha(B - B_{\mathrm{res}})}{\Delta B^2 + 4(B - B_{\mathrm{res}})^2} + \frac{\Delta B - \alpha(B + B_{\mathrm{res}})}{\Delta B^2 + 4(B + B_{\mathrm{res}})^2}\right] \tag{1}$$

This lineshape, often referred to as Dysonian, contains a parameter α ($0 \leq \alpha \leq 1$) indicating the mixture of absorptive and dispersive components of the susceptibility due to the finite depth of microwave penetration in a conducting sample. If the penetration depth is small in comparison to the sample grain size, α approaches 1.

Analysis of magnetic properties

Magnetic properties were examined using a Quantum Design (QD) Magnetic Property Measurement System in the temperature range of 1.8–300 K and under various applied magnetic fields. Samples were sealed in glass tubes to prevent reaction with air. Since magnetic measurements were performed on freshly grown samples with no exposure to air, we used the value of the saturated magnetization at the highest field and lowest temperature μ ($\mu_0 H = 7$ T$, T = 2$ K) to estimate the relative amounts of Eu^{2+} and Eu^{3+} in our samples. For a purely Eu^{2+} compound, we expect μ of 7 μ_B, so in general the valence is given by $x = \mu$ ($\mu_0 H = 7$ T$, T = 2$ K$)/7$ is the amount of Eu^{2+}, while the rest is $1 - x$ is the amount of Eu^{3+}. For our samples, the values are listed in Figure 2(a), ranging from 2.02 to 2.18. The inverse susceptibility was fit to the Curie-Weiss law with the values of the effective moment μ_{eff} and θ_W extracted to be 7.24 μ_{B} and 7.8 K (Sample 1); 7.37 μ_{B} and 11.7 K (Sample 2); 7.57 μ_{B} and 9.2 K (Sample 3); 7.85 μ_{B} and 6.4 K (Sample 4).

The specific heat data were collected on a QD Physical Property Measurement System from 0.4 K to 10 K and under various applied magnetic fields for Sample 1. Due to the high air-sensitivity of the $Eu_{11-x}Hg_{54+x}$ samples, they were covered with Apiezon N vacuum grease, the background contribution of which was subtracted. It was not, however, possible to measure electrical resistivity of $Eu_{11-x}Hg_{54+x}$, due to its high air-sensitivity, coupled with tendency to form mercury films on the crystal's surface, which then dominate electrical transport. The morphology of $Eu_{11-x}Hg_{54+x}$ crystals (see Figure 1 of the main text) did not allow for the implementation of the previous experimental solution for measurement of electrical resistivity, developed by our group.[4–6]

X-ray absorption measurements

HERFD XANES[7] spectra at the Eu L_3-edge were acquired at the ROBL beamline of the ESRF[8] on Sample 1. The incoming beam was monochromatized with a fixed-exit Si(111) double crystal monochromator. The Eu La1 characteristic fluorescence was analyzed with an X-ray emission spectrometer[9] based on Rowland geometry on which 4 Ge(333) crystal analyzers were mounted. The HERFD XANES were obtained by collecting the maximum of the Eu La1 emission line with a bandwidth of ~1.5 eV.

The sample was mounted on a sample holder in an Ar glovebox. The sample holder was sealed with a kapton tape to allow X-rays to enter and exit. The sample was sealed in a bottle and transported to the beamline. The bottle was open and the sample placed on the sample stage. Considering the time required to interlock the hutch and briefly scan the sample for centering, we estimate that the first HERFD XANES was acquired after 3 to 5 minutes after the sample holder was put in air. This time was unfortunately sufficient to oxidize a large amount of the Eu^{2+}.

Thermal analysis

Differential thermal analysis (DTA) was performed using a DSC 404C (NETZSCH) setup with a 10 K/min heating and cooling rate. Sample 1 was sealed in a Ta ampule (under argon, mass of $\sim$ 50 mg) and then measured in the temperature range from 25 °C to 500 °C. Results are summarized in Figure S2, with both heating and cooling curves showing a peritectic decomposition and incongruent melting of the $Eu_{11-x}Hg_{54+x}$ phase.

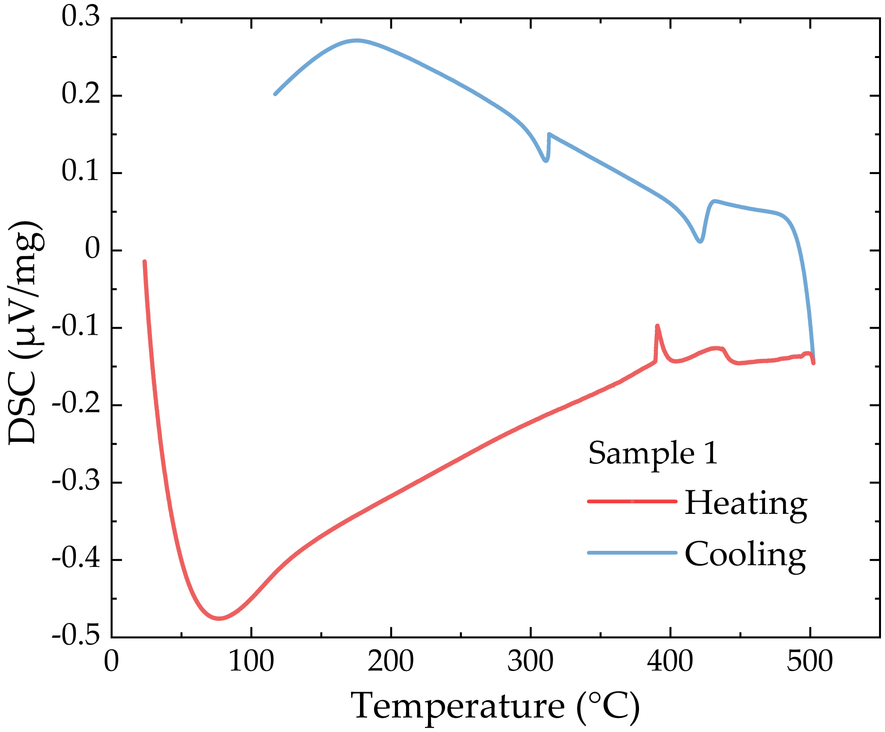

Figure S2: DTA of $Eu_{11-x}Hg_{54+x}$ (Sample 1) between 25 °C and 500 °C upon heating (red) and cooling (blue), both curves indicate peritectic decomposition and incongruent melting.

Single crystal data

Table S1: Crystallographic data for $Eu_{11-x}Hg_{54+x}$ (Sample 1)

Composition	$Eu_{10.24}Hg_{54.76}$
Space group	$P\overline{6}$
Pearson symbol	$hP65$
Formula units per unit cell, Z	1
Lattice parameters	
a / Å	13.6040(5)
c / Å	9.7744(5)
V / Å^3	1566.58(14)
Calc. density / g cm^{-3}	13.29
Crystal form	irregular shaped
Crystal size / μm	25 × 30 × 40
Diffraction system	RIGAKU AFC7
Detector	Saturn 724+ CCD
Radiation / λ/Å	MoKα, 0.71073
Scan; step / °; N(images)	φ, 0.6, 600
Maximal 2θ / °	59.88
Range in h, k, l	$-19 \leq h \leq 17$
	$-19 \leq k \leq 15$
	$-11 \leq l \leq 13$
Absorption correction	multi-scan
T(max)/T(min)	7.39
Absorption coeff. / mm^{-1}	143.59
N(hkl) measured	10551
N(hkl) unique	3146
R_{int}	0.064
N(hkl) observed	2926
Observation criteria	F(hkl) $\geq 4\sigma(F)$
Refined parameters	112
$R1$	0.037
$wR2$	0.075
Residual peaks / e Å^{-3}	$-2.21/2.62$

Table S2: Atomic coordinates and equivalent displacement parameters (in Å^2) in the crystal structures of $Eu_{11-x}Hg_{54+x}$ (Sample 1)

Atom	Site	x/a	y/b	z/c	U_{iso}
Eu1	$3j$	0.38991(17)	0.13055(17)	0	0.0172(4)
Eu2	$3j$	0.28359(16)	0.41101(16)	0	0.0170(4)
Eu3	$3k$	0.45731(16)	0.35867(17)	1/2	0.0170(4)
(Eu,Hg)4*	$2g$	0	0	0.3139(2)	0.0239(7)
Hg1	$6l$	0.50473(10)	0.39073(10)	0.15416(11)	0.0206(3)
Hg2	$6l$	0.43766(10)	0.12720(11)	0.33765(11)	0.0230(3)
Hg3	$6l$	0.27628(9)	0.21970(10)	0.23904(13)	0.0224(2)
Hg4	$6l$	0.05793(11)	0.24882(11)	0.20895(15)	0.0312(3)
Hg5	$6l$	0.15634(11)	0.69033(12)	0.33147(12)	0.0280(3)
Hg6	$6l$	0.09618(10)	0.47654(11)	0.16050(14)	0.0285(3)
Hg7	$3k$	0.23005(15)	0.07426(15)	1/2	0.0286(4)
Hg8	$3k$	0.16722(15)	0.25608(15)	1/2	0.0281(4)
Hg9	$3k$	0.09699(15)	0.45478(15)	1/2	0.0302(4)
Hg10	$3j$	0.13077(14)	0.11870(15)	0	0.0287(4)
Hg11	$2h$	1/3	2/3	0.1547(2)	0.0228(4)
Hg12	$2i$	2/3	1/3	0.3363(2)	0.0260(5)
Hg13	$1d$	1/3	2/3	1/2	0.0303(7)
Hg14	$1e$	2/3	1/3	0	0.0213(6)

*Occupancy by Eu and Hg is refined to 0.62(3):0.38

References

(1) Putz, H.; Brandenburg, K. Match 3! - Phase Analysis using Powder Diffraction. 2023.

(2) Joshi, J.; Bhat, S. On the analysis of broad Dysonian electron paramagnetic resonance spectra. J. Magn. Mag. Res. **2004**, 168, 284–287.

(3) Rauch, D.; Kraken, M.; Litterst, F. J.; Süllow, S.; Luetkens, H.; Brando, M.; Förster, T.; Sichelschmidt, J.; Neubauer, A.; Pfleiderer, C.; Duncan, W. J.; Grosche, F. M. Spectroscopic study of metallic magnetism in single-crystalline $Nb_{1-y}Fe_{2+y}$. Phys. Rev. B **2015**, 91, 174404.

(4) Witthaut, K.; Prots, Y.; Zaremba, N.; Krnel, M.; Leithe-Jasper, A.; Grin, Y.; Svanidze, E. Chemical and Physical Properties of YHg_3 and $LuHg_3$. ACS Org. Inorg. Au **2023**, 3, 143–150.

(5) Prots, Y.; Krnel, M.; Grin, Y.; Svanidze, E. Superconductivity in Crystallographically Disordered $LaHg_{6.4}$. Inorg. Chem. **2022**, 61, 15444–15451.

(6) Prots, Y.; Krnel, M.; Schmidt, M.; Grin, Y.; Svanidze, E. Uranium-mercury complex antiferromagnet: $UHg_{6.4}$. Phys. Rev. B **2022**, 106, L060412.

(7) Glatzel, P.; Weng, T.-C.; Kvashnina, K.; Swarbrick, J.; Sikora, M.; Gallo, E.; Smolentsev, N.; Mori, R. A. Reflections on hard X-ray photon-in/photon-out spectroscopy for electronic structure studies. J. Electron Spectrosc. Relat. Phenom. **2013**, 188, 17–25.

(8) Scheinost, A. C. et al. ROBL-II at ESRF: a synchrotron toolbox for actinide research. J. Synchrotron Radiat. **2021**, 28, 333–349.

(9) Kvashnina, K. O.; Scheinost, A. C. A Johann-type X-ray emission spectrometer at the Rossendorf beamline. J. Synchrotron Radiat. **2016**, 23, 836–841.
